# From iPSC towards cardiac tissue—a road under construction

**DOI:** 10.1007/s00424-017-2003-1

**Published:** 2017-06-01

**Authors:** Stefan Peischard, Ilaria Piccini, Nathalie Strutz-Seebohm, Boris Greber, Guiscard Seebohm

**Affiliations:** 1 Myocellular Electrophysiology and Molecular Biology, IfGH, Department of Cardiovascular Medicine, University Hospital Muenster, 48149 Münster, Germany; 2 Human Stem Cell Pluripotency Group, Max Planck Institute for Molecular Biomedicine, 48149 Münster, Germany; 3 Chemical Genomics Centre of the Max Planck Society, 44227 Münster, Germany; 4 Innovative Medizinische Forschung (IMF), Münster, Germany; 5 Institut für Genetik von Herzerkrankungen (IfGH), Department für Kardiologie und Angiologie, Universitätsklinikum Münster, 48149 Münster, Germany

**Keywords:** Cardiac differentiation, Induced pluripotent stem cell, Myocyte physiology, Signaling pathway, Long QT syndrome

## Abstract

The possibility to generate induced pluripotent stem cells (iPSC) opens the way to generate virtually all cell types of our human body. In combination with modern gene editing techniques like CRISPR/CAS, a new set of powerful tools becomes available for life science. Scientific fields like genotype and cell type-specific pharmacology, disease modeling, stem cell biology, and developmental biology have been dramatically fostered and their faces have been changed. However, as golden as the age of iPSC-derived cells and their manipulation has started, the shine begins to tarnish. Researchers face more and more practical problems intrinsic to the system. These problems are related to the specific culturing conditions which are not yet sufficient to mimic the natural environment of native stem cells differentiating towards adult cells. However, researchers work hard to uncover these factors. Here, we review a common standard approach to generate iPSCs and transduce these to iPSC cardiomyocytes. Further, we review recent achievements and discuss their current limitations and future perspectives. We are on track, but the road is still under construction.

## Introduction

Our heart beats a lifetime with astonishing precision and adapts to stress situations to increase heart rate (positive chronotropic) and pump load (positive inotropic). The cardiac rhythm is controlled by the sino-atrial node (SAN). Preloading of the heart with blood is efficiently allowed by the atria, whereas cardiac ejection work is due to about 85% to ventricular function. To study the physiology, pathophysiology, and pharmacology of cardiac cells, it would be highly desirable to have a source of pure human cell populations of the SAN, atria, and ventricles. However, natural sources like biopsies or non-transplantable hearts are rare. Recent advances in induced pluripotent stem cells (iPSC) and gene editing techniques hold the promise to fill this gap.

A decade ago, Kazutoshi Takahashi and Shinya Yamanaka showed that a set of transcription factors can reprogram somatic cells to acquire a pluripotent stem cell state [57]. With these well-designed experiments, they paved the ground for a new scientific field, i.e., iPSCs. It became clear that cell identity is much more plastic than previously imagined. IPSCs provide a cellular basis for novel approaches for translational as well as disease- and pharmacology-oriented research. Targeted differentiation of IPSCs provides researchers with defined cells of a given type. In combination with genome editing and specific 2D/3D cell culture systems, an iPSC-based system has emerged that represents an alternative to classical studies utilizing model organisms.

In the past years, several controversies could be solved. These included genome stability, which was shown to be similarly stable as in other cell lines. Furthermore, effects of the origin of the reprogrammed cells, resulting in a partially maintained epigenetic memory, could be eliminated by optimized protocols and technical procedures. IPSC clone to clone variability was observed and might represent the variability inherent to the genetic variance of somatic cells from one organism.

The reprogramming procedures started with usage of integrating retroviral vectors and excisable lentiviral and transposon-based vehicles. More recently, non-integrating episomes and RNA-based systems like mRNA-transfections and Sendai virus vectors have been used. Methods exclusively depending on application of small-molecule compounds or protein transduction have been partially successful and are under further development. The key transcription factors for reprogramming towards pluripotency are sex determining region Y-box 2 (SOX2), Kruppel-like factor 4 (KLF4), octamer-binding transcription factor 4 (OCT4), and cellular myelocytomatosis (cMYC). Additional factors have been studied as so-called enhancers [56]. Using optimized protocols, almost all cell types form higher organisms can be reprogrammed to result in iPSCs.

Among other cell types, iPSCs were early on transduced towards cardiac cells. Here, we review current knowledge on iPSC maintenance and cardiac induction towards functional cardiac cells. Moreover, we comment on current shortcomings and future perspectives of iPSC-derived cardiac cells.

## iPSC maintenance is regulated by members of the TGFβ-superfamily

The cultivation of iPSC over a long period of time has improved significantly over the past decade. Just a few years back, iPSCs and other stem cell types were seeded and kept on feeder cell layers like inactivated mouse embryonic fibroblasts (iMEF), which supported the self-renewal and proliferation of the desired stem cells [3, 17, 32, 63, 67]. This technique was known as the most effective way to keep stem cells undifferentiated, but it was not efficient due to low stem cell density and the need to remove unwanted fibroblasts from the actual stem cells in every replating step [63].

Therefore, a lot of effort was taken to invent alternatives for feeder-based stem cell culture to increase the effectivity and efficiency of stem cell maintenance. The most promising way to achieve this goal was the invention of feeder-free stem cell culture systems under defined conditions. The idea behind the feeder-free cultivation of stem cells was to understand the role of feeder cells concerning self-regeneration and proliferation and to mimic the effects in feeder-free systems.

In the past few years, various feeder-free maintenance protocols were published using different media compositions and supplements. Regarding the effect of feeder cells, all protocols can be cut down to basal media containing three major components: FGF-2, Activin-A, and TGFβ-1 [3, 14, 17, 33]. These members of the TGFβ superfamily are interacting with feeder and stem cells and activate the self-renewal machinery in iPSC and human embryonic stem cells (hESC) [65].

The self-renewal of stem cells is in the first place controlled via expression of the pluripotency genes encoding NANOG, Oct-4, and Sox2, which can be activated by the before mentioned factors [3, 7, 15, 17, 65].

In feeder cell-based cultures, i.e., iMEF feeder layers, FGF-2 interacts with the feeder cells. Only in the presence of FGF-2, the iMEFs are turned into a supportive feeder layer for stem cells preventing their spontaneous differentiation. FGF-2 activates the secretion of TGFβ-1, Activin-A, and Gremlin by the iMEFs [3, 15, 65], which then interact with the stem cells seeded on top, inducing self-renewal and proliferation [14, 17]. TGFβ-1 and Activin-A induce the Smad2/3 signaling pathway, which in the end, enhances the expression of NANOG—a strong pluripotentcy factor. Gremlin works, however, as a potent bone morphogenic protein (BMP) inhibitor, preventing spontaneous differentiation regularly induced by smad1/5/8 activation [14, 15, 18, 28, 33, 65].

**Fig. 1:**
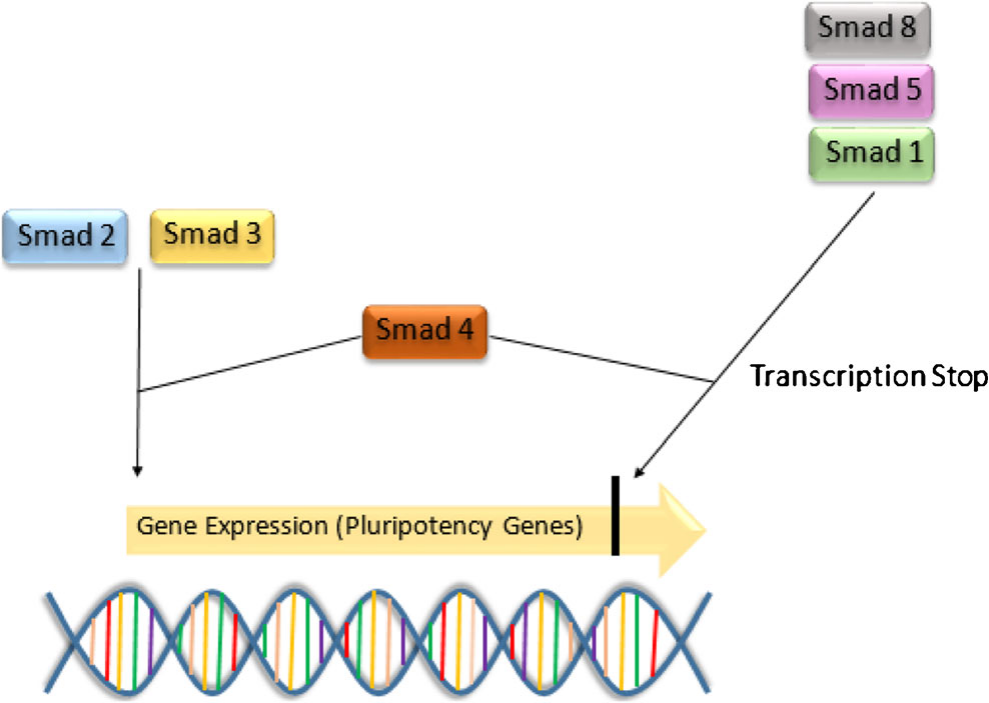
Smad-signaling pathways. Smad 2/3 in cooperation with Smad 4 evoke the expression of pluripotency genes such as NANOG, while the presence of Smad 1/5/8 blocks the expression of pluripotency genes when Smad 4 is present

In feeder-free stem cell culture systems, the addition of FGF-2 alone is not sufficient to sustain pluripotency in hESC or iPSC, due to a missing feeder cell layer. Thus, the secretion of Activin-A and TGFβ-1 has to be mimicked by adding these factors in an appropriate concentration. The addition of Activin-A and TGFβ-1 activates the same smad2/3-signaling pathway in hESC and iPSC [17, 19, 28]. TGFβ-1 and Activin-A both bind to their type-II receptors ActIIB or TGFbIIB located on the stem cell surface [15, 19, 65]. Binding of the ligand to its type-II receptor activates a signaling cascade: the type-II receptor phosphorylates the type-I receptors, which in turn activate the receptor activated by smad (R-Smad) [15, 65]. TGFβ-1 and Activin-A activate the R-Smad called Smad2/3, while BMP-4 is activating Smad1/5/8 [7, 15, 19, 28, 65]. Both Smads then bind to smad 4, resulting in a formation of a complex. The R-smad/Smad4 complex binds to other transcriptional factors in the cell nucleus and, thereby, controls the expression of genes like pluripotency factors or BMP [15, 19, 65] (Fig. 1).

Both smad2/3 and smad1/5/8 can interact with the proximal NANOG promoter. The binding of smad2/3 is strong in hESC and mostly sustained by TGFβ-1 [19, 65], while smad1/5/8 is weak in hESC, but steadily increases by BMP induction and leads to differentiation of hESCs [15, 17, 33, 65].

**Fig. 2:**
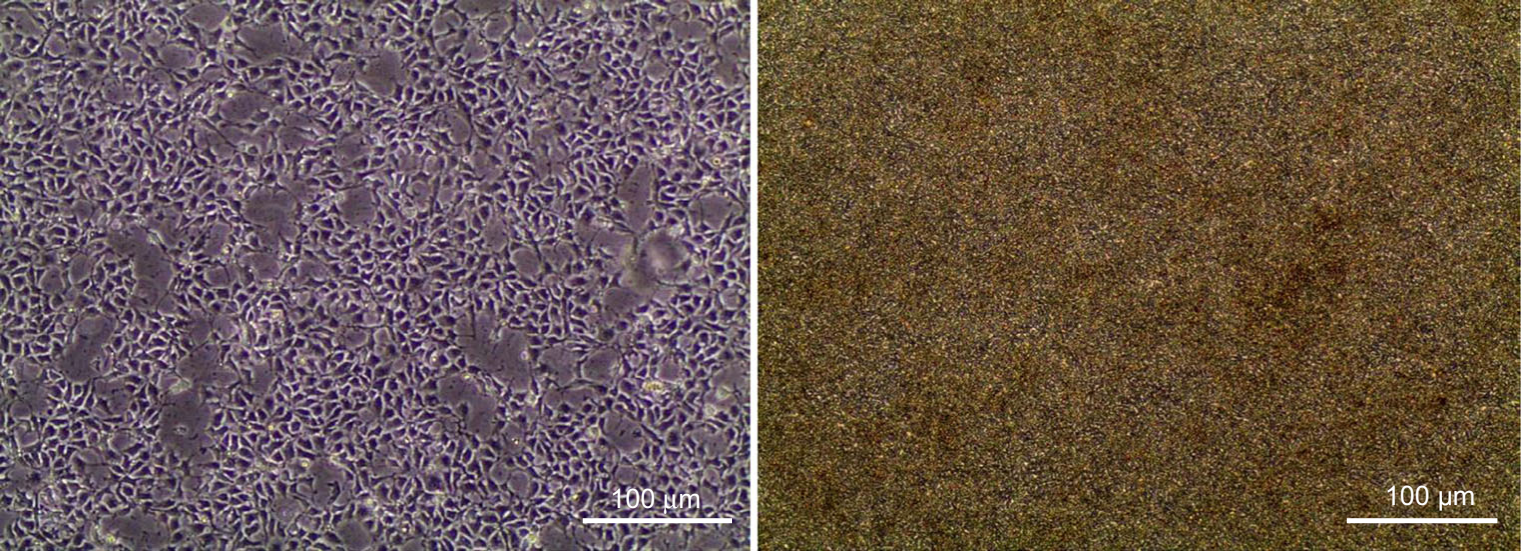
Stem cell line SFS.1 used for cardiac differentiation in optimal conditions. *Left*: SFS.1 at day 1 after replating. The cell layer is already dense and the cell morphology is vital. *Right*: SFS.1 at day 4 after replating. Cells reached confluency and individual cell bodies cannot be distinguished. Cells are ready for the start of the differentiation

Not only the activation of smad1/5/8 triggers spontaneous differentiation, but also the inhibition of smad2/3, which shows its important role in sustaining pluripotency. Greber et al. showed that blocking of the smad2/3 pathway with SB431542 drastically decreases NANOG within 12 h leading to spontaneous differentiation [17, 18, 29, 65]. The importance of the smad2/3 pathway is also underlined by the fact that many ligands are able to activate this pathway to increase the expression of NANOG [17]. Not only TGFβ-1 and Activin-A can induce the smad2/3 pathway, but also the autocrine signal of Nodal which helps hESC to sustain pluripotency [18, 65] (Fig. 2).

## Roles of the Wnt/BMP pathway in pluripotency and differentiation

The control over the Wnt and BMP pathways in stem cell culture is crucial for a stabilized maintenance and controlled differentiation of target cells [7, 17]. As mentioned before, the BMP pathway gets controlled in feeder-cell-based stem cell culture systems via expression of Gremlin. Gremlin is a potent inhibitor for the BMP pathway and, thus, for the activation of smad1/5/8 [3, 15, 65].

In feeder-free conditions, the BMP pathway has to be blocked by defined chemicals at moderate concentrations. An effective factor for BMP-inhibition is Dorsomorphine, which is stable under cell culture conditions and does not affect the cell growth or viability. Anyways, it is important to use Dorsomorphine at the right concentration [14, 29]. It was shown that a low inhibition of BMP leads to autocrine expression of more BMP and, thereby, to differentiation and mesodermal specification of hESC and iPSC. A too effective inhibition of BMP leads to neural induction and ectodermal specification [1, 15, 17].

**Fig. 3:**
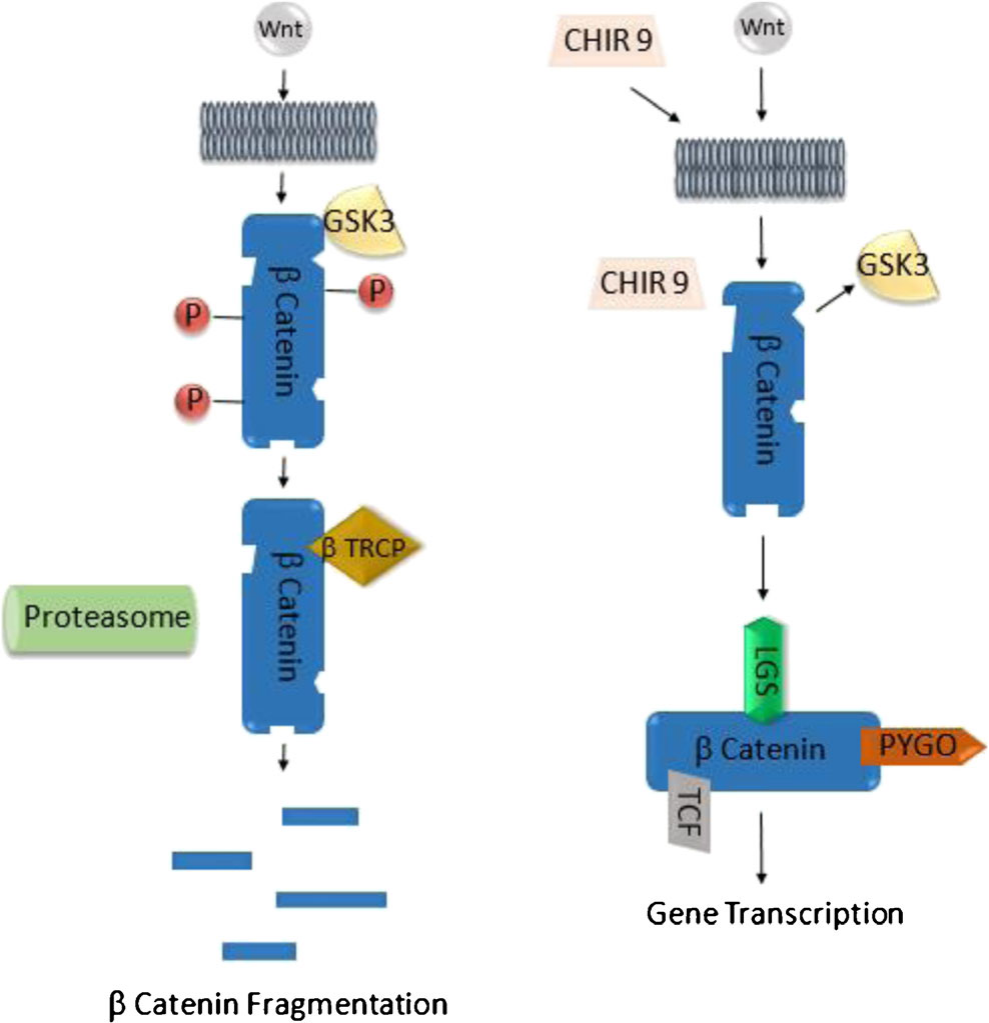
Activation of WNT signaling by CHIR is crucial for differentiation. *Left*: Wnt binding to β Catenin is blocked by GSK3, suppressing the Wnt pathway and inhibiting gene transcription. *Right*: The Wnt activator CHIR acts as a GSK3 inhibitor leading to Wnt/β Catenin interaction with subsequent gene transcription

The control of the Wnt pathway in stem cell maintenance is not as crucial as the control of BMP, as long as the cells grow as expected. Most of the time, stem cells keep their pluripotency when the smad2/3 pathway is active and BMP is appropriately blocked [65]. Nevertheless, even small changes in culture conditions evoke changes in gene expression and spontaneous differentiation [17]. This differentiation can usually be detected at an early stage, while changes in a colonies phenotype become striking. One of the main reasons for spontaneous differentiation, if BMP is blocked, is the unwanted activation of the Wnt pathway. By blocking the Wnt pathway with chemicals like C59, IWP-2, IWR-1, or XAV-939, the differentiation can be stopped [1, 28]. Anyways, the constant block of Wnt by one of the before mentioned chemicals is not recommended, while it was shown that the efficiency of cardiac differentiation (and probably of more cell types) is negatively affected by elongated Wnt inhibition (Fig. 3).

During cardiac differentiation, BMP and Wnt need to be precisely controlled at specific time points to enhance the efficiency of cardiomyocyte differentiation [3, 69].

**Fig. 4:**
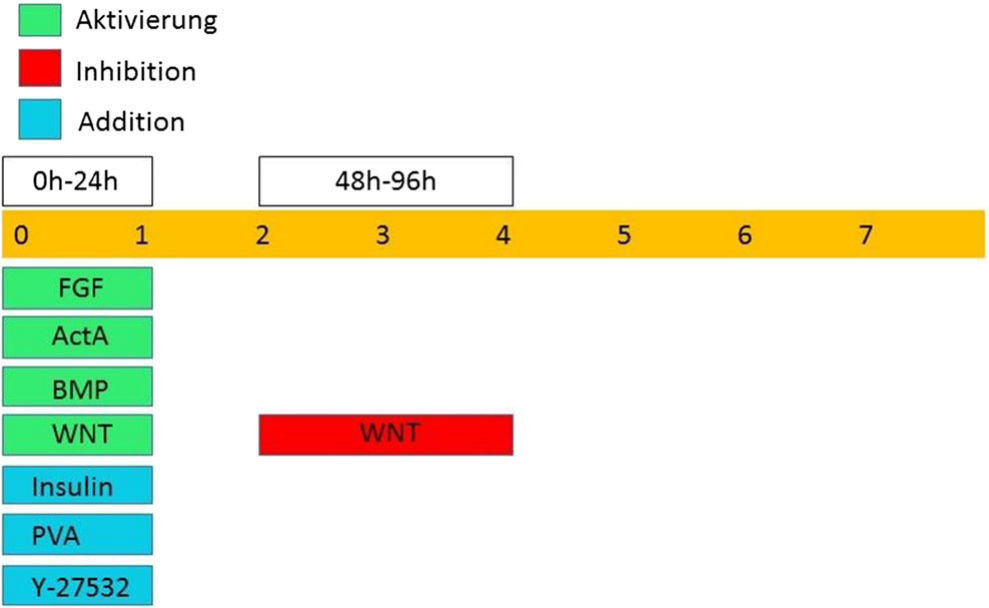
Principle of cardiac differentiation. Most important is activation of the Wnt and BMP pathway from hour 0–24 and the Wnt inhibition from 48 to 96 h after initiation of differentiation

Figure 4 illustrates the basic principle of an efficient cardiac differentiation. On the day of cell seeding, cells are kept in a basal medium containing insulin, FGF-2, CHIR-9, BMP, and in some protocols Activin-A [3, 33, 69]. Obviously, BMP is given to activate the BMP pathway via smad1/5/8 to induce the spontaneous differentiation of the seeded stem cells [65]. Together with CHIR and Insulin, an upregulation of mesodermal markers is achieved and mesodermal specification of the seeded cells is for example indicated by enhanced expression of SOX2 [15, 69]. The addition of Activin-A is beneficial for mesodermal specification and can promote the cardiac differentiation in many cell lines. Additionally, the Rock-Inhibitor Y-27532 is included in the medium to reduce apoptotic cell signals, which increases cell survival [1, 17, 69]. FGF-2 is given at day 0 to the differentiation medium because it represses early neural induction by down regulation of the expression of PAX-6, an early neuronal marker [15, 17].

A controlled cell survival is crucial for an efficient cardiac differentiation, because the seeded cell number and its distribution have huge effects. Cells have to be seeded very densely, almost over-confluent, and their distribution has to be as homogeneous as possible to ensure uniform tissue development [69].

On day 1 after seeding, the medium is exchanged for a basal medium containing ascorbate and transferrin/selenium to give the cells time to recover from the first differentiation step and to potentially increase the cardiac differentiation efficiency by activating the MEK/ERK pathway [7, 30, 33, 35]. This basal medium is used throughout the differentiation process. If the 24-h BMP and Wnt induction on day 1 was followed precisely and the cell distribution is consistent, there is just one crucial step towards a successful cardiac induction: on day 2 and 3 after cell seeding, the Wnt pathway needs to be blocked by chemicals like C59 or IWP-2 [1, 3, 28] to block the smad2/3 pathway and to induce the expression of early cardiac and mesodermal markers like ISL-1 and NKX 2.5 [7, 30, 33, 35, 65]. This triggers the cardiac specification followed by maturation in basal medium for another 5–6 days. During this maturation, the cells begin to express further cardiac markers like cTNT or α-Actinin [7, 28, 29, 33, 67, 68]. On day 8 after seeding, a beating monolayer is produced containing cardiac cells with more than 98% purity.

If the purity of these early cardiomyocytes is not suitable, which is reflected by a high percentage of non-beating areas, there are two options to increase the concentration of cardiac cells. If the beating areas can easily be distinguished from non-cardiac tissue, a simple dissection of the desired region followed by separation and replating is the technique of choice. Usually, the replating of cardiac cells is done on a mixture of matrigel and gelatine [14, 28, 67].

If the desired cells are mixed with other, undefined cell types, a dissection is rather impossible. In this case, a difference in cell metabolism between muscular and non-muscular cells can be used for selection. Muscular tissue is able to change its metabolism from glycolysis to lactate cycle, which other mesodermal cell types cannot accomplish. By changing the normal basal medium to a medium that contains lactose instead of glucose, all non-muscular cells lose their ability to keep up their energy household and run into apoptosis. The cells, which are still vital, are necessarily muscular cells containing a high amount of cardiac cells, due to differentiation specificity [28, 69].

The general principle of efficient cardiac induction was postulated by Brüstle et al. [28], showing the work flow of all cardiac differentiation protocols used by research groups all over the world.

## The principle of cardiac induction

In the early phase of differentiation, a descent control of the BMP-4 and Wnt pathway is crucial for directed cardiac outcome [28, 65]. In the so-called induction phase, the pluripotent cells get pushed towards a mesodermal fate. The outcome is the efficient production of mesodermal progenitor cells, given that the BMP-4 and Wnt pathways were activated in an adequate level. Because the BMP-4 pathway is self-regulated, the addition of BMP-4 to the differentiation medium is sufficient to activate the pathway. For the activation of Wnt, transcription factors like CHIR9 are used [3, 28, 69]. The mesodermal specification can be confirmed by the expression of early mesodermal markers like T-Brachyury. The addition of insulin to the medium enhances the expression of T-Brachyury and was shown to increase cardiac differentiation efficiency [7, 15, 18, 28, 30, 35].

The second phase, the so-called specification phase, is characterized by the manipulation of the Wnt pathway and its inhibition. After 1 day of recovery in medium lacking transcription factors, the mesodermal progenitor cells are treated with a Wnt inhibitor, for example C59, leading to the specification of cardiac progenitor cells. If done correctly, a high output of cardiac cells can be achieved. The cardiac specification is characterized by upregulation of later mesodermal markers like ISL-1 and early cardiac markers as Nkx2.5 [7, 28, 30, 33, 65].

The enrichment phase is characterized by maturation of the cardiac progenitor cells into cardiac tissue. From this stage, no transcription factors need to be given to the medium. The trans-differentiation into cardiac tissue happens automatically over the following week, resulting in a autonomously beating tissue [35] expressing cardiac markers like cTnT or α-Actinin [7, 28, 29, 33, 68]. This tissue is in an early state, homologous to early embryonic heart tissue, and it is not very structured. It contains a mixture of different types of cardiac cells, including ventricular, nodal, or pacemaker cells [23]. The beating in the early cardiac cells is regulated by L-Type Ca^2+^channels expressed in the young tissue [1, 20, 23, 32, 35, 64] in contrary to mature tissue where the heartbeat is manipulated by other ion channels like KCNQ1 [68, 69].

**Fig. 5:**
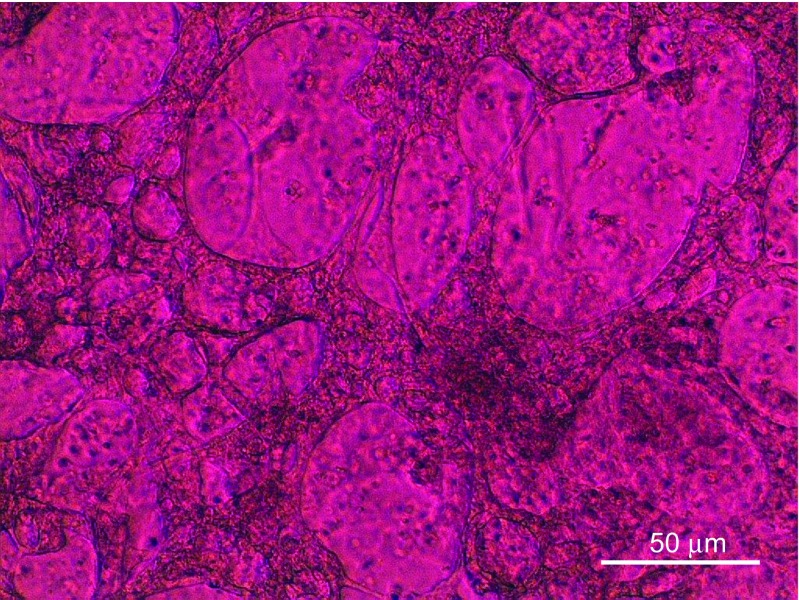
Differentiated cardiac tissue on day 8 of trans-differentiation. Morphological changes from stem cell to cardiac like cell. Autonomously active beating muscular tissue expressing cardiac markers and producing action potentials

In later stages of cardiac differentiation, mainly after 4–6 weeks of maturation, the tissue changes its ion channel composition, replacing the L-Type Ca^2+^channels with KCNQ1 and others resulting in a more synchronous and stronger beating of the tissue [1, 20, 23, 32, 68, 69] (Fig. 5).

### Alternative targets for cardiac induction—EOMES as cardiac inducer

The before mentioned manipulation of the Wnt and BMP pathway for cardiac induction is an efficient procedure to produce cardiac tissue expressing cardiac markers and ion channels, but it is not the only possibility available. The controlled expression of a transcription factor called EOMES, which takes influence of T-box genes, leads to similar results [11, 15, 28, 48].

Early experiments in *Xenopus* embryos by Ryan et al. in 1996 [50] showed a strong influence of EOMES on heart development. By blocking the natural expression of EOMES by injecting EOMES-engrailed into the developing embryos, changes in heart development were achieved. The changes ranged from mildly hypoplastic to vestigial to totally heartless, suggesting an important role of EOMES in early cardiac induction [50].

Ryan et al. also showed that EOMES works in a dose-dependent manner during mesoderm development in *Xenopus*, mouse, and zebrafish. Low expression of EOMES leads to the development of more ventral-mesodermal tissue, while high dosages of EOMES induce the production of dorsal-mesodermal tissues like muscle or notochord. These findings strongly implicated that EOMES might also have a crucial role in human heart development [50].

Indeed, EOMES was found to play a strong role in human heart development as it is also conserved as a T-box gene in the human genome [6, 11, 50].

In 2012, Jelle van Ameele [61] published a study concerning EOMES expression during cardiac differentiation of ESC, which proved the suggested role of EOMES in cardiac development. It was found that EOMES directly induces the expression of the transcription factor Mesp1 within 24 h, suggesting a binding of EOMES directly to the Mesp1 promoter [6, 10, 11, 45, 61]. Genomic analyses showed three conserved T-box regions (T1, T2, T3) where T2 is not conserved in mammalians responding to EOMES binding. Especially, T3 proved as strong enhancer for Mesp1 expression when EOMES is present [61].

The same study showed that MESP1 expression leads to the production of early cardiac markers like cTnT, as well as GATA4 and 6, NKX2-5, TBX20, HAND2 [7, 29, 30, 33, 45, 61, 65, 67]. At the same time, endodermal markers as Sox17 and FOXA and neural markers like b-Tubulin III, Sox-1 [7, 15, 18, 29], and Pax6 are downregulated, ensuring the promotion of cardiovascular cell formation.

MESP1 is expressed from day 2 to day 4 in the developing embryos heart and was shown to be the most important marker for mesodermal specification [24, 45, 69]. From day 4, MESP1 is not expressed anymore and is not detectable in later differentiation or in the adult heart [61].

The EOMES-induced expression of MESP1 was shown to be controlled with the help of a before mentioned transcription factor, Activin-A [61]. Experiments showed that high levels of Activin-A lead to the reduced expression of MESP1 usually induced by EOMES [6, 48]. It was even shown that the expression of mesodermal markers like cTnT and CD31 is negatively influenced by Activin-A in a dose-dependent manner [61]. On the other hand, the presence of Activin-A leads to the expression of endodermal markers as Sox17 [7, 15, 18] and a-fetoprotein [61]. Cell culture experiments have proven the negative effect of Activin-A on cardiac induction by the reduction of beating areas in differentiated tissue and a resulting changed, non-cardiac phenotype. Thus, Activin-A acts as a regulator for EOMES recruitment [61].

An alternative transcription factor playing a similar role as EOMES is Brachyury. Brachyury, a T-box transcription factor, is expressed in early mesoderm before cardiac specification [7, 15, 18, 30, 69]. Its activity is overlapping with EOMES and it also leads to MESP1 expression, because it binds to the T-box elements. Thereby, it is cooperating with EOMES towards cardiac induction [24]. Nevertheless, both EOMEs and Brachyury [10, 48] are necessary to achieve an efficient cardiac formation in stem cell differentiation. It surely is possible to derive cardiac cells by just activating a part of the necessary pathways, but the yield of cardiac cells in the culture increases when many pathways are activated in the proper manner.

Traditionally, cardiac maturation has been shown by the expression of a few markers like cTnT. As cardiac induction is a very complex event, usage of more than one characteristic gene is suggested. Recently, genome wide analyses revealed a plethora of genes that may be used as readout providing sufficient insight into the process of cardiac induction and maturation [47].

### Electrical equivalent circuits are well-suited to describe developmental induction processes

**Fig. 6:**
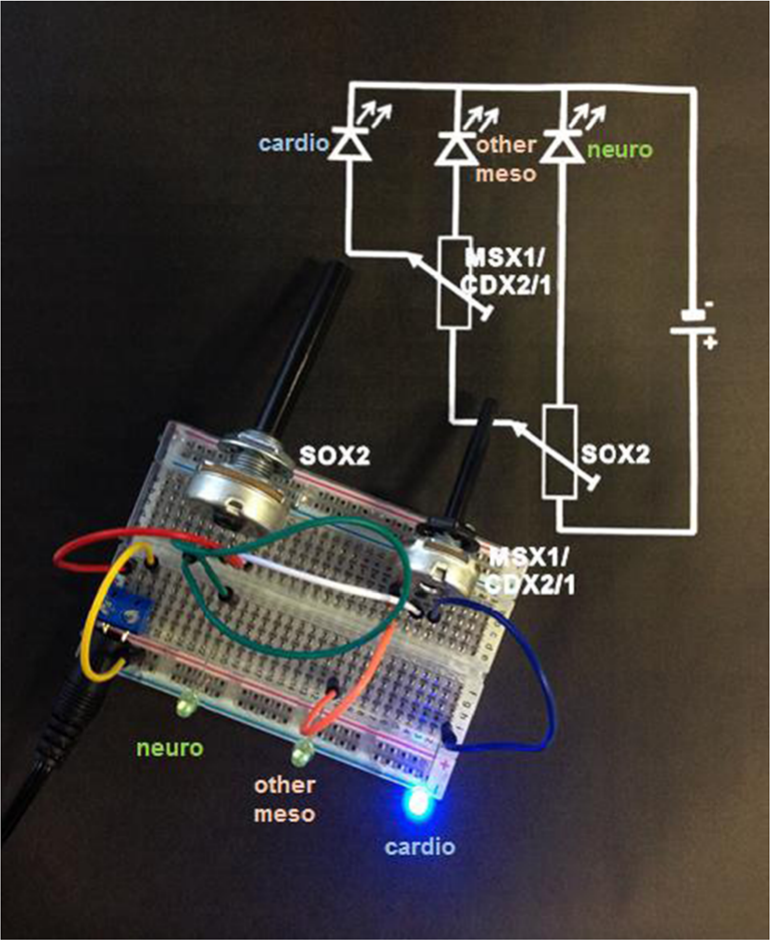
Mechanisms to drive stem cells into a specific lineage are well compared to an electrical circuit diagram. Growth factor stimuli applied in cascade can be compared to control dials in an electrical circuit. Switching specific dials shifts the driving force for differentiation from one line to the other. Resistive dials are better suited to describe the biological processes rather than simple on/off switches. The increase in resistance in one electrical path increases currents in the alternative path and thereby determine which light emitting diode (LED) will glow. Here, *blue* represents cardiac cells (cardiac lineage), other mesodermal cells (other mesodermal lineage) are indicated by *pink*, and neurogenic cells (neuronal lineage) are indicated by *green*

Induction of specific lineages by modulation of pathways has been described traditionally by charts, in which pathways were inter-connected by lines and arrows. However, these charts can easily be misinterpreted. Biology processes are of analogous nature, allowing for graded responses rather than for digital on/off answers. Cardiac induction is under control of key signals that set the course [48]. Here, we introduce the concept of using electrical equivalent circuits. To describe the induction of cardiac differentiation tunable switches allowing for graded settings are required. Usage of simple potentiometers (poti = variable resistors) enable such a behavior. A simple example is given in Fig. 6.

## Cardiac myocyte function

The adult human heart harbors a plethora of different cells including cardiomyocytes that are functionally highly heterogeneous within and among the conduction system, the atria, and ventricles. Furthermore, connective tissue and complicate the picture. In order to have valuable cell models for the individual cell types, subtype-specific trans-differentiation giving rise to pure cell types would be very valuable. However, most commonly used cardiomyocyte protocols produce a mixture of pacemaker cells, atrial-like, and ventricular-like cells, whereas the ventricular-like cell population is the largest and pacemaker cells are the fewest. For disease modeling, pharmacology and physiology as well as for analysis of individual patients, the purification of the mentioned cell types is required. Recently, Chen et al. described the generation of iPSC clones that express genetically encoded voltage-sensitive dyes under control of cardiac cell type-specific promotors allowing for analyses of, e.g., atria-like cells in a mixtures of phenotypically mixed iPSC-cardiomyocytes [8, 55]. Whereas some problems are solved by this approach, pure cell populations for specific biochemical, genetic, and pharmacological are required to address several scientific questions. Protocols to derive the major types of cardiomyocytes have been described. Sinus node-like pacemaker cells can be enriched by specific protocols and second step selection processes [27, 31]. Zhang et al. reported in 2011 that retinoid signaling is a key for specification of atrial-like vs. ventricular-like cardiomyocytes during cardiac differentiation of human ESCs [70]. Retinoid acid receptor activation significantly decreased cardiac differentiation efficiency, but increased the proportion of atrial-like cardiomyocytes up to almost pure atria-like cells. Supposedly, enrichment is achieved by increased apoptosis of non-atrial-like cardiomyocytes. In agreement with this hypothesis, application of Noggin and/or retinoid acid receptor antagonists significantly increased the cardiac differentiation and led to efficient cardiomyocyte differentiation and about 83% of cells with characteristic ventricular-like action potentials. A detailed overview of protocols has been given by Talkhabi et al. [58]. Thus, it is possible to obtain almost pure subtypes of iPSC-derived cardiomyocytes that can be used for subtype-specific experiments.

IPSC-cardiomyocytes derived directly from healthy and diseased persons have been successfully used to study disease phenotypes. Clearly, the gross of studies predicted phenotypes that often had been studied for years in simpler and more robust cell systems. Indeed, a combination of the traditional robust expression systems like mammalian cell cultures and/or *Xenopus laevis* oocytes together with iPSC-derived cardiomyocytes tends to produce the most convincing reports. However, recent studies provided results that clearly benefited from the unique features of the iPSC system. Over the last years, iPSC-cardiomyocytes have been used to investigate the molecular mechanisms of diseases like long QT syndrome (LQT) and other heart diseases.

## Myocyte physiology, disease modeling, and pharmacogenetics

Positive inotropic effects in the context of the physiological acute-stress reaction via β-adrenergic pathways are mediated by the SAN. The SAN pacemaker cells show a spontaneous rhythmic activity without reaching a stable resting membrane potential. Main inducer of this auto-rhythmicity of SAN cells is the depolarizing hyperpolarization-activated current If (I_f_ = funny current, also named hyperpolarization current I_h_ or queer current I_q_) and to lesser extent, voltage-gated calcium currents I_Ca_. The human I_f_ current is carried by the hyperpolarization-activated and cyclic nucleotide-gated channels HCN4. In response to stress-associated β-adrenergic stimulation, cyclic AMP (cAMP) is generated which binds directly to HCN4 and increases I_f_ by shifted voltage-dependent activation. The enhanced I_f_ current speeds up SAN cell depolarization and, thereby, the heart rate. Typically, pace-making cells in iPSC-derived cardiomyocytes have a prominent I_Ca_ but small I_f_, which is different from typical adult human SAN cardiomyocytes. It has to be kept in mind that in contrast to isolated adult human SAN cells, spontaneous activity of beating iPSC-cardiomyocytes may be more dependent on Ca^2+^ currents than on I_f_ [27]. This difference is important in the context of disease modeling and pharmacology. Nevertheless, Jung JJ et al. succeeded to model the disease “sick sinus syndrome” that is based on dysfunctional HCN4 channels [27]. Furthermore, such iPSC sinus node-like cells may hold some potential in sinus node specific pharmacology [2]. In order to increase I_f_ (*HCN4*), transgenic expression of HCN4 may proof helpful [51]. Interestingly, spontaneous activity of such nodal-like cells shows some degree of rate variability similar to the heart rate variability in adult humans, promising a potential use for complex physiological experimentation and disease modeling [5, 37].

Positive inotropic effects during acute physiological stress are due to β-adrenoreceptor-initiated cAMP-mediated activation of protein kinase A (PKA) and phosphorylation of target proteins in atrial as well as ventricular cardiomyocytes. Of particular importance is the parallel phosphorylation and associated activation of calcium I_Ca,L_ and potassium currents I_Ks_. The balanced activation of I_Ca,L_ and its antagonizing I_Ks_ protects from excessive action potential lengthening and potentially lethal arrhythmias. Indeed, imbalanced I_Ks_/I_Ca,L_ currents are believed to trigger *torsade de pointes* arrhythmias in the long QT 1/5 syndrome (LQT1/5, characterized by pathopysiologically reduced I_Ks_) and timothy syndrome (also named LQT8, characterized by pathopysiologically increased I_CaL_). The QT interval characteristically lengthened in all long QT syndromes is largely dependent on ventricular electrical events. In order to utilize iPSC-cardiomyocytes to sufficiently understand events in LQTS, a uniform ventricular cell population is required. The first study to analyze LQT syndrome in patient-derived cardiomyocytes was published by Moretti et al. and paved the ground for a series of further studies [42]. In recent years, several long QT syndromes—in part combined with highly complex modifier situations—have been modeled in iPSC-cardiomyocytes [4, 34, 36, 43, 46, 49, 54, 66, 68]. Classically, long QT syndromes have been relatively simple to explain and electrophysiological techniques allowed to show the functional alterations. Therefore, long QT syndrome studies have been fruitful and have been chosen as first disease entities to be studied in iPSC-cardiomyocytes. However, several other cardio-pathological conditions could be modeled [12]. These include catecholaminergic polymorphic ventricular tachycardia (CPVT), dilated cardiomyopathy (DCM), hypoplastic left heart syndrome and hypertrophic cardiomyopathy, Marfan syndrome, Barth syndrome, Leopard syndrome, and Friedreich ataxia [25]. Especially the cardio-pathological conditions associated with cellular structural aberrations can be difficult to tackle because the cell morphology of iPSC-cardiomyocytes is clearly different from an isolated adult cardiac myocyte.

IPSC-cardiomyocytes have been used to model complex pharmacological events with given genotype resulting in drug-induced LQT syndrome [25, 59]. On the contrary, pharmacological I_K_ activation in iPSC-cardiomyocytes and genotype specific pharmacologic rescue of LQTsyndrome has been described recently [41, 52, 62, 68]. Thus, iPSC-cardiomyocytes are valuable in pharmaco-genetic research as well. However, expression of I_Ks_ is very time dependent. Long differentiation times of at least 4 weeks are required to allow for detection of I_Ks_. Even after this relatively long period, expression is low and suitability of iPSC-cardiomyocytes to model LQTS1 characterized by I_Ks_ defect have been discussed [9, 40]. A second ion current that is clearly reduced in stem cell-cardiomyocytes compared to adult human cardiomyocytes is the inward rectifier current I_K1_ [16]. Similar to I_Ks_, I_K1_ increases with maturation. An approach to obtain stem cell-derived cardiomyocytes that have controlled I_Ks_ or I_K1_ and can be used for example in pharmacological studies is the usage of genetically engineered iPSC cell lines carrying the ion channel gene under control of an inducible promoter [26].

Triggered by the electrical event (action potential) at the myocyte surface, Ca^2+^ is allowed to enter the cardiac cell via L-type calcium channels (conducting I_Ca,L_). These channels are in physical contact with ryanodine receptors (Ryr2). The increased Ca^2+^ influx via I_Ca,L_ and their structural rearrangements associated with channel activation stimulates ryanodine receptors that in turn release large amounts of Ca^2+^ from the sarcoplasmic reticulum (SR), a process called calcium-induced calcium release. The Ca^2+^ released from the SR binds to troponin to trigger the muscular cross bridge cycle, allowing for the generation of contraction force. All reports on contraction force of iPSC-cardiomyocytes report much smaller forces developed by these cells compared to that of native human cardiomyocytes. As the internal structure of iPSC-cardiomyocytes is much less ordered compared to adult cardiomyocytes, it can be expected that contraction force generated by iPSC-cardiomyocytes is much less directed. In other words, the force vectors point to different directions and, thus, sum up to a much smaller degree compared to adult cardiomyocytes with their highly ordered sarcomeres. In addition, the regions showing ordered sarcomere structures occupy much less in volume of the iPSC-cardiomyocyte compared to adult cardiomyocytes, allowing for reduced total force per volume. Both phenomena may explain the main proportion of reduced iPSC-cardiomyocyte force. Several cultural techniques are under the development to improve the performance of the iPSC-cardiomyocytes [13]. These include chronic electrical stimulation and β-adrenoreceptor activation to allow for more efficient competent sarcomere development [13, 22, 60]. Nevertheless, iPSC-cardiomyocytes have been used to address questions of active force development. Most of the passive reset force in cardiomyocytes is provided by titins. Recently, iPSC-cardiomyocytes have been successfully used to model a titin-associated disease [21]. Mechanical studies benefit from recent advances in 3D cell culture that mimic several aspects of heart tissue. Mannhardt et al. lately made a huge step towards a functional 3D cardiomyocyte culture [38]. The group was able to realize a strip-format, force generating engineered heart tissue (EHT) cultivated in agarose casting molds. Research on these EHT showed an improved cardiac phenotype compared to cardiomyocytes derived from 2D cultures and a more realistic drug response. Nevertheless, the observation of cell organelles like the sarcoplasmatic reticulum indicate a still not fully maturated cell state comparable to cardiomycytes of a newborn [38].

The cardiac current I_to_ is conducted by Kv4.3/KChIP channels. Human mutations in KChIP can lead to gain-of-function of channels and cause early-onset of persistent lone atrial fibrillation [44]. The I_to_ current exerts a marked functional transmural gradient along the cardiac wall, which is believed to be important for cardiac function and to be protective against arrhythmias. The I_to_ gradient is enabled via expression of differently spliced KChIP β–subunits in the Kv4.3/KChIP complex through the hearts wall [53]. In order to perfectly model the disease of early-onset of persistent lone atrial fibrillation it would be required to transdifferentiate to pure ventricular cell types reflecting transmural gradient to sufficiently model KChIP gradient. Ideally, this would be in a cardiac wall mimicking 3D cell culture. However, such a scenario is not yet achieved.

## Benefits of iPSC-derived cardiomyocytes for regenerative medicine

The native human cardiac myocyte does not exert significant regeneration after an infarct. Therefore, the hope of replacing dead cardiac tissue after infarction with patient-specific iPSC-based cardiac tissue was borne out when the first reports of iPSC-cardiomyocytes were published. Initial studies showed in animal models that iPSC-cardiomyocytes could be integrated into infarcted tissue. However, the ensuing functional effects were marginal and the benefit was only very weak. However, since these early trials, reports of improved protocols have been published and nowadays iPSC-derived cardiac tissue can be engineered and it was recently integrated in infarcted heart tissue. Weinberger et al. showed that implantation of human hEHT strips integrated successfully, showed cardiomyocyte proliferation and vascularization. Moreover, they provided evidence for electrical coupling to the intact heart tissue in engrafted hearts and for a clear function improvement (31%). Masumoto H et al. reported clearly beneficial effects by implantation of a combination of iPSC-derived cardiomyocytes, endothelial cells, and vascular mural cells into infarcted, immune tolerant rat hearts, which induced both vascularization and myocardial function improvement [39]. Together, these studies demonstrate that human heart muscle constructs can repair vasculature and myocardium in the injured heart. Some problems have not been sufficiently addressed and solved. These include potential generation of teratogens by the iPSC-derived cells and destruction of exogenous iPSC-derived cells by the host immune system. Still, the results promise that in far future, heart-associated morbidity and mortality could be reduced.

In summary, the iPSC field exploded in a short period of time. Given the dramatic pace of development in the cardiac iPSC field, it is likely that iPSC-derived cardiac cells will foster basic cardiac research, pharmacology, pharmacogenetics, and even clinics.

## Abbrevations

cTnT: Cardiac troponin-T
EOMES: Eomesodermin
FGF-2: Fibroblast growth factor-2
hESC: Human embryonal stem cells
KChIP: K-channel interacting proteins
Mesp1: Mesoderm posterior basic helix-loop-helix transcription factor 1
Oct-4: Octamer-binding transcription factor 4
PAX-6: Paired-box-gene-6
Smad: Mothers against decapentaplegic homolog
Sox2: Sex determining region Y-box 2
TGFβ-1: Transforming growth factor beta 1
Wnt: Wingless-related integration site
